# Associations between circulating metabolites and pca: a bidirectional two-sample Mendelian randomization study

**DOI:** 10.1007/s12672-025-03204-9

**Published:** 2025-07-18

**Authors:** Jing Lv, Yonghong Deng

**Affiliations:** 1 Hunan Traditional Chinese Medical College, Zhuzhou, China; 2 Health Management Center, The Second Xiangya Hospital, Central South University, 139 Renmin Middle Road, Changsha, 410011 Hunan People’s Republic of China

**Keywords:** Prostate cancer, Metabolites, Mendelian randomization

## Abstract

**Background:**

Prostate cancer (PCa) remains the most prevalent cancer among male globally. Despite the critical role of genetic factors in PCa pathogenesis, recent advances in metabolomics have highlighted the significant contributions of circulating metabolites to genetic risk profiles for PCa. However, the causal relationship between metabolites and PCa is not yet unclear.

**Methods:**

We utilized a bidirectional two-sample Mendelian randomization (MR) approach, analyzing metabolite datasets from the Canadian Longitudinal Study of Aging (CLSA), the Cooperative Health Research in the Region of Augsburg (KORA) study, and the TwinsUK study and PCa dataset from the Oncoarray. Replication analyses were performed with the UK Biobank. Instrumental variables (IVs) were selected based on established MR criteria and analyzed using methods including the Wald ratio, inverse-variance weighted (IVW), MR-Egger, and weighted median. To ensure robustness, sensitivity analyses were performed using Cochrane’s Q, Egger’s intercept, MR-PRESSO, and leave-one-out (LOO) methods.

**Results:**

We identified causal relationships between circulating metabolites and PCa risk. After removing high influential SNPs and outliers and reanalysis, we obtained the levels of N6-carbamoylthreonyladenosine (OR 0.61, 95% CI 0.37–1.01, *p* = 0.054) and 4-ethylphenylsulfate (OR 0.66, 95% CI 0.47–0.92, *p* = 0.015) causally associated with PCa. All results passed FDR correction; 4-ethylphenylsulfate also remained significant after Bonferroni adjustment. Reverse MR analysis highlighted robust causal relationships of PCa to homovanillate (OR 1.07, 95% CI 1.03–1.10, *p* = 5.49 × 10 − 5) and X-12,627 (OR 1.03, 95% CI 1.01–1.04, *p* = 7.54 × 10^−5^) levels.

**Conclusion:**

These insights underscore the etiology and risk factors of PCa, providing genetic evidence for the development of therapeutic targets and contributing to elucidating disease mechanisms, suggesting potential diagnostic biomarkers.

**Supplementary Information:**

The online version contains supplementary material available at 10.1007/s12672-025-03204-9.

## Background

Prostate cancer (PCa) is prevalent globally and ranks among most commonly diagnosed malignancies in men, contributing significantly to cancer-related deaths [1, 2]. Epidemiological studies have underscored that PCa is closely related to age, race, obesity, genetics, lifestyle, and Metabolic reprogramming [3]. Metabolic reprogramming including glycolysis, glutamine metabolism, and lipid metabolism had significant changes in during the progression of PCa, which were closely related to the occurrence and metastasis of PCa [4, 5]. Metabolic reprogramming is a key factor in the occurrence and development of PCa, correlating closely with cancer occurrence and metastasis [6]. Therefore, as the end products of organismal metabolic processes, changes in the level and composition of metabolites could reflect pathological state and have the potential to elucidate the etiology of PCa.

In recent years, changes in metabolites have gained significant attention in the early diagnosis and pathophysiological study of PCa. Several studies have demonstrated that specific metabolic alterations can reflect the onset and progression of PC. Xu et al. conducted a non-invasive serum metabolomic analysis and identified significant changes in several metabolites, including phosphatidylcholine (PS[16:0/20:2]) and Carnitine [C14:0], in PCa patients, highlighting their potential clinical application as biomarkers for PCa diagnosis [7]. Further multivariate regression analysis also indicated that the metabolic panel could distinguish between PCa patients and healthy controls with high diagnostic performance [8]. Additionally, Schmidt et al. identified significant differences in PC and amino acid levels in the serum of PCa patients, reinforcing the potential of metabolites as early biomarkers for PCa [9]. Moreover, researchers have found significant differences in metabolites like Tenascin C (TNC) and Apolipoprotein A1V (Apo-AIV) in the serum of PCa patients, suggesting a strong association with cancer metastasis and prognosis [10]. The development of metabolic panels that track these metabolic alterations could offer an effective tool for early PC detection and monitoring treatment response. However, these studies are not without limitations. Serum metabolites are influenced by a range of factors, including the body’s physiological state, coexisting diseases, and environmental conditions. As a result, it is challenging to rule out confounders, making it difficult to establish definitive causal relationships between specific metabolites and PCa. This complexity highlights the need for further research to control for these factors and validate the true associations between metabolites and PCa.

Mendelian randomization (MR) offers a powerful framework for assessing causal relationships between exposures and diseases. By using genetic variations as instrumental variables (IVs), which are randomly distributed in the population and established early in life, unaffected by environmental factors or diseases, MR reduces confounding biases inherent in traditional observational studies, providing more accurate causal inferences [11]. Employing a two-sample MR approach allows us to mitigate the constraints tied to evaluating exposure and outcome in the same cohort, thus boosting the statistical robustness. This method also reduces the risk of the winner’s curse, a common issue in single-sample MR analyses [12]. Moreover, bidirectional analysis allows us to better explore the causal relationships between exposure and outcomes, avoiding reverse causality [13].

Hence, we utilized a bidirectional two-sample MR approach, analyzing metabolite datasets from the Canadian Longitudinal Study of Aging (CLSA), the Cooperative Health Research in the Region of Augsburg (KORA) study, and the TwinsUK study and PCa dataset from the UK Biobank and Oncoarray. This rigorous analysis aimed to robustly assess the causal relationship between circulating metabolites and the risk of PCa.

## Materials and methods

### Overview

**Fig. 1:**
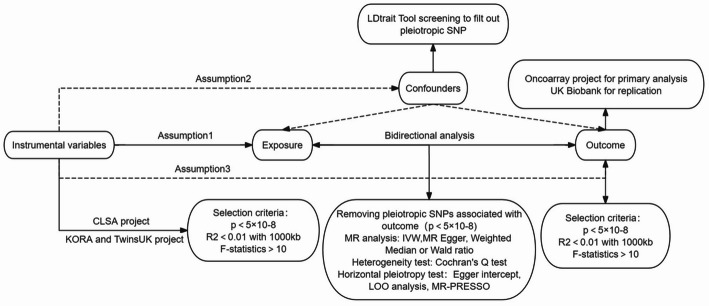
The flowchart of the bidirectional two-sample mendelian randomization study

Our study adheres to the guidelines for strengthening mendelian randomization research reports (STROBE-MR). The overall design is illustrated in Fig. 1 [14].

### GWAS data sources

We utilized the two large serum metabolite datasets: the Canadian Longitudinal Study of Aging (CLSA), the Cooperative Health Research in the Region of Augsburg (KORA) study, and the TwinsUK study as exposures. These metabolites were categorized based on their super-pathways and sub-pathways as described in the original studies (Table S1). In the primary study, we used summary genome-wide association study (GWAS) data from the OncoArray project with PCa as outcome. In replication studies, we utilized summary GWAS data from the UK Biobank, which encompasses populations from across the UK. For detailed information on these datasets, refer to the Supplementary materials.

### Selection of instrumental variables (IVs)

In the analysis of circulating metabolites-PCa associations, the correlation threshold of 5 × 10^−8^ was established to extract SNPs that are strongly associated with circulating metabolites. Next, to ensure the independence of IVs, the R^2^ < 0.01 and the clustering window was set to 10,00 kb to exclude SNPs with linkage disequilibrium. Furthermore, F-statistics were calculated to exclude the weak IVs (F-statistics > 10) [14].

### MR analysis

For the MR analysis, the alignment of SNP data between exposure and outcome was facilitated using the “harmonise_data” function in the “TwoSampleMR” R package [15, 16], with the exclusion of palindromic sequences. SNPs not directly matchable were replaced by high LD proxies (R2 > 0.8) as identified by the 1000 Genomes Project. SNPs without appropriate proxies were excluded. We applied the Wald ratio method for single SNP phenotypes, and for multiple SNPs, the fixed effects inverse-variance weighted (IVW) method was used unless heterogeneity tests suggested significant variance (*p*<0.05), in which case the random effects IVW method was adopted as our primary analysis tool. This method aggregates Wald ratios from each SNP assuming validity but is vulnerable to pleiotropy bias. Hence, additional methods like MR-Egger and weighted median analyses were utilized to validate causality and assess estimate consistency under varying assumptions.

### Sensitivity analysis and directional test

In sensitivity analyses, heterogeneity among SNPs was assessed using Cochrane’s Q test (*p* < 0.05 indicated significant heterogeneity), and horizontal pleiotropy was evaluated through the Egger intercept, MR-PRESSO test, and leave-one-out (LOO) analysis. The validity of potential candidate metabolites was affirmed by consistent directional outcomes across three MR methods, absence of pleiotropy, and no outliers in LOO analysis. The Steiger test was employed to exclude reverse causation bias, indicating no disproportionate SNP influence on PCa relative to metabolite levels (Steiger *p* > 0.05).In addition to the primary analyses, reverse MR analyses were conducted to investigate the effects of breast cancer as an exposure on circulating metabolites.

### Confounders investigation

While a suite of statistical methods was utilized in our sensitivity analyses to evaluate potential violations of MR assumptions, we further delved into potential confounders through the LDlink website (https://ldlink.nih.gov/?tab=ldtrait), and the R² and LD range were set as 0.01 and 1000 kb, respectively. Our aim was to ascertain if SNPs linked to circulating metabolites were concurrently associated with other genetic confounding factors (Age, family history, baldness, height, physical activity, diets, smoking, prostatitis and prostate size, autoimmune diseases, diabetes) that might skew MR estimates. If these SNPs showed associations with any of these potential confounding factors, we proceeded to discard these SNPs and the outliers mentioned in MR PRESSO and LOO analysis and then repeated the MR analysis to ensure the robustness of our findings.

### Statistical analysis

All MR estimates were reported as odds ratios (ORs) with corresponding 95% confidence intervals (CIs). To account for multiple comparisons, both the Bonferroni correction and the Benjamini-Hochberg false discovery rate (FDR) method were applied to adjust p-values within each super-pathway and sub-pathway. Although these two methods differ in their underlying principles—Bonferroni being more conservative and FDR allowing greater sensitivity—a consistent statistical significance threshold of 0.05 was applied to both, in order to facilitate interpretability and comparability of the results across correction methods. Meta-analyses utilized both common and random effects models to synthesize findings from Oncoarray and UK Biobank employing restricted maximum likelihood to estimate heterogeneity, expressed as Tau², and I² statistics to quantify the proportion of total variation due to heterogeneity. All computations were executed in R (version 4.3.2) with the TwoSampleMR, MendelianRandomization, ieugwasr, mr.raps, meta and plinkbin packages.

## Results

### Characteristics of the included IVs

After validating the efficacy, we extracted 4068 SNPs, with each metabolite represented by 1 to 22 SNPs. The average R2 was 1.67% (ranging from 0.36 to 71.82%), and the average F-statistic was 111.25 (ranging from 27.45 to 2297.79), indicating that all SNPs are sufficiently strong instruments for MR analysis (F-statistic > 10, Table S2).

### Main MR analysis results

Under FDR-adjusted significance thresholds, we identified significant causal relationships between the genetic determinants of 16 circulating metabolites and PCa risk within super-pathway-based analyses (Table S3). Similarly, 39 circulating metabolites demonstrated significant causal effects on PCa within sub-pathway-based analyses (highlighted in yellow). Additionally, we conducted analyses using the MR-Egger and weighted median methods on metabolites with three or more IVs, aimed at verifying the robustness of the causal inference. The results showed that the vast major of the effects of metabolites on PCa were consistent in direction across different methods(Table S4), except for the effect of the levels of X-21,312 and N-acetylcarnosine (show in yellow highlighted).

### Replication analysis

We conducted a replication analysis using UK Biobank. Under FDR-adjusted thresholds, we identified significant causal associations between 6 circulating metabolites and PCa in super-pathway-based analyses (Table S5), and between 16 circulating metabolites and PCa in sub-pathway-based analyses (highlighted in yellow). The MR-Egger and weighted median methods identified inconsistencies in the direction of effects of ibuprofen levels (Table S6, show in yellow highlighted). Summarily, the causal relationship between the levels of N6-carbamoylthreonyladenosine (N6CA), 4-ethylphenylsulfate (4-EPS), 4-vinylphenol sulfate and the risk of PCa have been validated by the UK Biobank (Table S7).

### Sensitivity analysis

The vast major of the causality did not show any heterogeneity or pleiotropy, except for the effect of X-11,381 levels in Oncoarray dataset, and the effect of N-acetyl-aspartyl-glutamate levels in UK Biobank (Table S8-S9, show in yellow highlighted), which were verified by funnel plots and scatter plots (Figure S1–-S4). MR PRESSO identified rs144885738 as an outlier in UK Biobank. In the LOO analysis, we observed a shift in the impact on PCa when excluding some SNPs (Figure S5-S6), suggesting that the MR results were strongly driven by these SNPs. Through the Steiger test, we further validated the causal direction of these metabolites to PCa.

### Confounders investigation and reanalysis

**Fig. 2:**
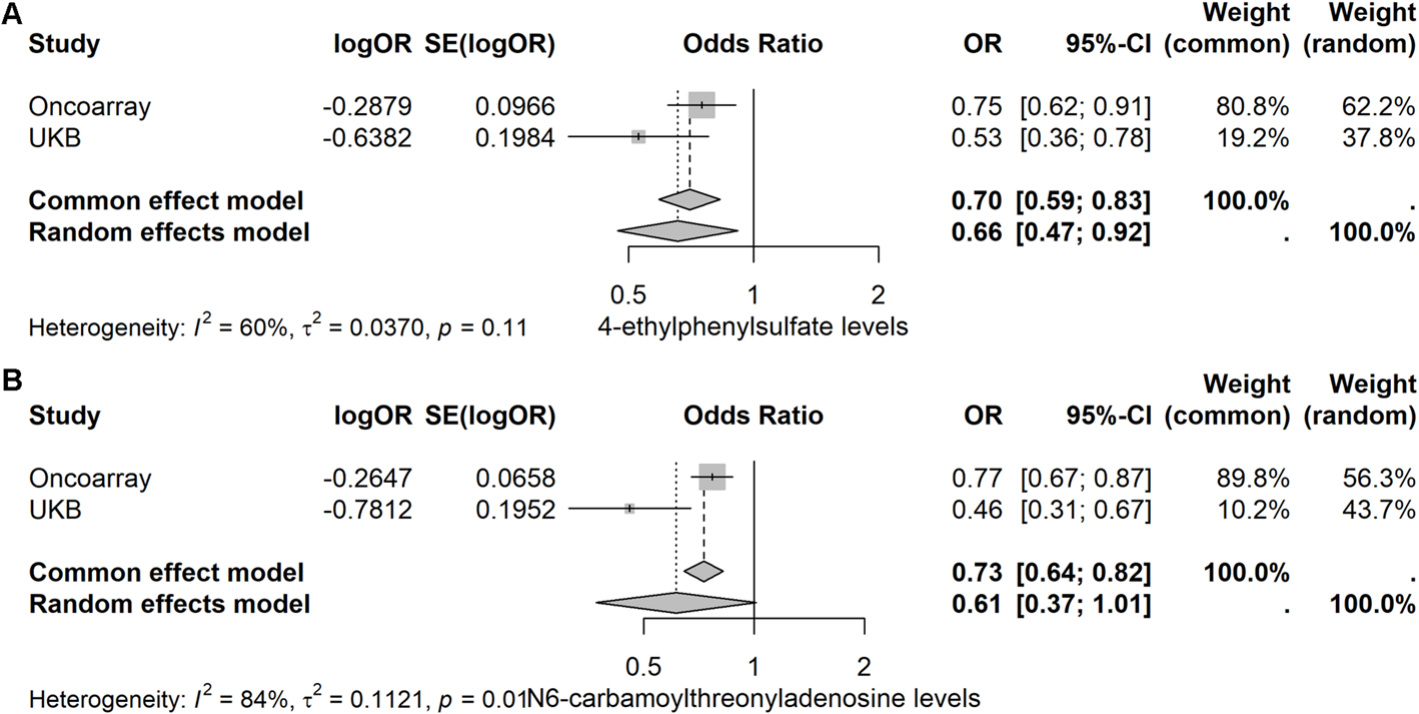
Significant negative causal relationships between N6-carbamoylthreonyladenosine and 4-ethylphenylsulfate levels and PCa

Using LDlink, we identified pleiotropic SNPs that are related to potential confounders (Table S10). By combining these with outliers identified in previous LOO analyses, we excluded a total of 16 IVs exhibiting horizontal pleiotropy or high-influential SNPs (Table S11). After reanalysis, we eliminated metabolites with inconsistent results across the main MR methods (show in yellow highlighted). In the Oncoarray dataset, PCa was ultimately identified with 35 causally associated metabolites (Table S12). In the UK Biobank, PCa was ultimately identified with 15 causally associated metabolites (Table S13). When intersecting the results of the two datasets, we identified two metabolites potentially associated with PCa. For N6CA, the odds ratio (OR 0.61, 95% CI 0.37–1.01, *p* = 0.054) suggests a potential association with PCa. 4-EPS demonstrated a statistically significant association with PCa (OR 0.66, 95% CI 0.47–0.92, *p* = 0.015, Fig. 2, Table S14). Notably, 4-EPS remained statistically significant after Bonferroni correction (*p* < 0.05 in both datasets; Table S3 and S5), indicating a robust association. In contrast, N6CA was not statistically significant under Bonferroni adjustment in the UK Biobank dataset (*p* = 1.00, Table S5).

**Reverse MR analysis**.

**Fig. 3:**
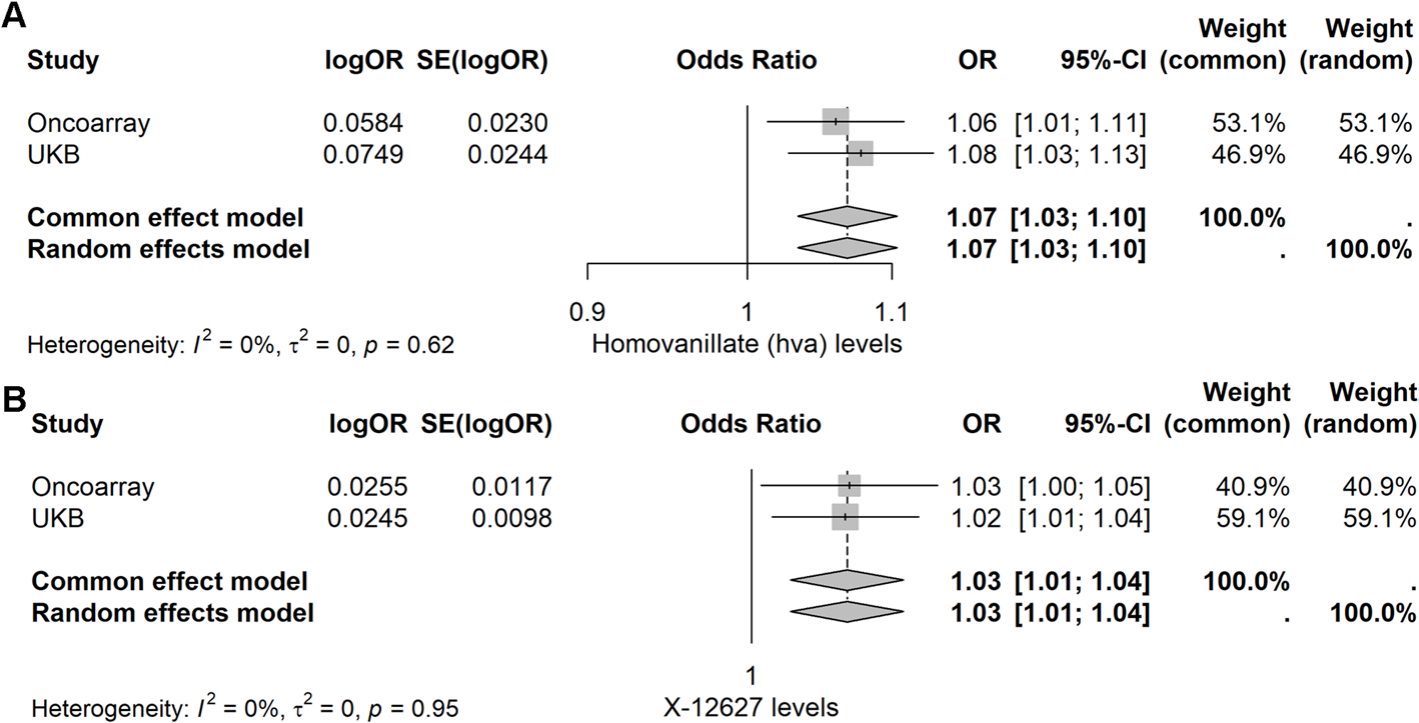
Significant positive causal relationships between homovanillate (HVA) and X-12,627 levels and PCa

For reverse analysis, we extracted 187 SNPs (Table S15). PCa were found to have causal relationships with 136 and 68 metabolites in Oncoarray and UK Biobank dataset, respectively (Table S16, 17, show in yellow highlighted). The results of different MR methods showed inconsistency in the effect of PCa on some specific metabolites (Table S18-19, show in yellow highlighted). We further intersected the results of Oncoarray and UK Biobank and ultimately obtained homovanillate (HVA, OR 1.07, 95% CI 1.03–1.10, *p* = 5.49 × 10 − 5) and X-12,627 levels (OR 1.03, 95% CI 1.01–1.04, *p* = 7.54 × 10^−5^) with robust causal relationships with PCa (Fig. 3, Table S20, show in yellow highlighted).

## Discussion

PCa imposes a substantial global health burden, and identifying new biomarkers, treatment targets, and clarifying its etiology had the great significance [17]. In this study, we utilized the large-scale GWAS of circulating metabolites and PCa to systematically identify whether metabolites are the cause of PCa through the bidirectional two-sample MR study. The MR analyses demonstrated that the levels of 4-EPS (OR 0.66, 95% CI 0.47–0.92, *p* = 0.015) causally associated with the risk of PCa. Reverse MR analysis highlighted robust causal relationships of PCa risk to HVA and X-12,627 levels. Given the high dimensionality of metabolomics data, we applied both the FDR correction and the Bonferroni correction to adjust for multiple testing. FDR is widely used in omics studies due to its superior power to detect true positives while controlling the proportion of false discoveries, making it more suitable for exploratory analyses involving hundreds of metabolites [18]. In contrast, Bonferroni correction is more conservative, effectively reducing the risk of false positives but potentially overlooking biologically relevant signals. In our analysis, we adopted FDR as the primary correction strategy for metabolite-wide screening, while Bonferroni correction was used in the final stage to validate key findings under more stringent significance criteria [19]. This dual correction strategy enhances both the discovery potential and the statistical robustness of our results.

It is worth noting that in the causal association analysis between N6CA and PCa, although the odds ratio (0.61, 95% CI 0.37–1.01) suggests a potential inverse association, the p-value was 0.054, which did not meet the conventional threshold for statistical significance (*p* < 0.05). Moreover, this association did not remain significant after Bonferroni correction, further suggesting the possibility of a chance finding. In Mendelian randomization studies, limited numbers of instrumental variables (IVs) can reduce statistical power, making it difficult to detect modest causal effects [20]. In this analysis, only two IVs were available, which may restrict the robustness of the results and weaken the strength of causal inference. Additionally, random variation and measurement error may also contribute to marginal significance [21]. Further studies with larger sample sizes and additional independent IVs are warranted to validate this finding.

To our knowledge, there has been no research on the association between these four metabolites and PCa. 4-EPS is a tyrosine-derivative. 4-EPS have been associated with reduced health and hyperlipidemia, and may be the mediator of periodontitis leading to pre-diabetes [22, 23]. At present, the most extensively explained function of 4-EPS was closely related to neurological disorders including autism spectrum disorder and anxiety [24–27]. However, the relationship between 4-EPS and cancer, including PCa, is still unclear. This study first discovered a causal relationship between 4EPS and PCa. 4-EPS, a derivative of tyrosine, is synthesized via specific enzymatic pathways involving gut microbiota processes, including tyrosine ammonia lyase, phenolic acid decarboxylase, vinyl phenol reductase enzymes, and host sulfotransferase [24]. The gut microbiota has been increasingly recognized for its role in modulating host physiology and potentially contributing to disease states, including PCa. Research has linked alterations in gut microbiota composition, particularly changes in *Firmicutes* and *Bacteroidetes*, to conditions such as immune dysregulation and cancer, including PCa progression [28, 29]. The metabolites of gut microbiota can regulate the host androgen level and induce benign prostate hyperplasia, chronic nonbacterial prostatitis, and PCa occurrence [30]. These studies proved that 4-EPS may serve as a key intermediary linking gut microbiota activity to PCa. Understanding this relationship could offer insights into new therapeutic targets or biomarkers for PCa prevention or treatment.

In addition, HVA which is the main metabolite of dopamine can reflect the dopamine level [31]. Previous studies demonstrated that dopamine inhibited the proliferation and cancer stem cell-like cell of PCa cell [32, 33]. Notably, HVA expression was abnormal which has been shown to be acted as biomarker for breast cancer and neuroblastoma [34, 35]. Hence, this suggests that HVA regulates the malignant functions of PCa through its involvement in dopamine pathways. Next, N6CA has been associated with several health conditions beyond PCa. Serum level of N6CA was positively associated circulating interleukin-6, complication of type 2 diabetes mellitus, and hypertension [36–38]. In addition, N6CA had relationship to renal function injury, chronic kidney disease, and ESRD development [39–42]. While its direct role in PCa has not been extensively studied, its associations with inflammatory markers and metabolic disorders suggest potential implications for PCa biology, particularly in the context of systemic inflammation and metabolic dysfunction. Notably, the role of X-1245 remains largely unexplored in scientific literature, especially concerning its potential associations with health conditions or diseases such as PCa.

Forward MR analyses can uncover the etiology and risk factors of diseases, providing genetic evidence for the development of therapeutic targets. Whereas, reverse MR analyses contribute to elucidating disease mechanisms, thereby suggesting potential diagnostic or prognostic markers. In our study, in addition to some recognized metabolites with potential diagnostic or prognostic value that deepen our understanding of the metabolic mechanisms of PCa, other metabolites with unknown functions provide valuable insights for the development of new biomarkers and further metabolomics research. Our study has illuminated several metabolites with causal associations to PCa, yet numerous questions remain. Future research should expand the range of metabolites analyzed to uncover additional associations and elucidate the specific biological mechanisms through which these metabolites influence cancer development. Longitudinal studies are also proposed to observe changes in metabolite levels over time and their correlation with cancer risk and progression. Additionally, integrating environmental and lifestyle data could help clarify the interplay of genetics, metabolism, and external factors in cancer etiology. Finally, clinical trials designed to modulate metabolite levels could provide new insights into effective prevention and treatment strategies, potentially leading to improved outcomes for PCa patients. These efforts will not only fill the existing gaps but also deepen our understanding of the metabolic underpinnings of PCa.

This study has significant strengths. Firstly, the study is based on a specific ethnic group, which may limit the generalizability of findings to other populations. Genetic and environmental factors can vary significantly between ethnicities, impacting metabolite profiles and disease risk. Additionally, it employs reverse MR analysis to delve deeper into the causal relationships between metabolites and PCa. This study has several limitations that should be considered when interpreting the results. First, the research is limited to a European population, which may restrict the generalizability of the findings to other ethnic groups. Second, this study lacks data on the different stages and PSA levels of PCa, which are crucial for understanding how metabolic changes relate to cancer progression. Lastly, while the study identified two circulating metabolites associated with the risk of PCa, the exact regulatory mechanisms behind these metabolites remain unclear. Further research is required to confirm their role and understand how they contribute to the metabolic changes that drive PCa development.

## Conclusion

N6CA and 4-EPS, identified through forward MR analysis, are likely contributing to the etiology of PCa. Conversely, HVA and X-12,627, derived from reverse MR analysis, offers valuable insights for diagnosis and prognosis in PCa. Further research is warranted to clarify this finding and understand the underlying biological mechanism linking these metabolites to PCa pathogenesis.

## Electronic supplementary material

Supplementary Material 1

Supplementary Material 2

Supplementary Material 3

## Data Availability

This study exclusively used publicly available data, which was approved by the ethical reviews of the studies cited in the Methods section.
