# Supplementary material for: Associations between circulating metabolites and pca: a bidirectional two-sample Mendelian randomization study
Source: Discov Oncol. 2025 Jul 18;16:1370. doi: 10.1007/s12672-025-03204-9 (PMC12274190; doi:10.1007/s12672-025-03204-9)
Supplement: Supplementary file 1 — Supplementary Material 1 [file 12672_2025_3204_MOESM1_ESM.docx]

**Appendix 1**

**The Canadian Longitudinal Study of Aging (CLSA)**

This study examines 8,299 unrelated individuals of European ancestry, aged between 45 and 85 at the time of recruitment. The participants were genotyped using the Affymetrix Axiom genotyping platform, and genotype imputation was performed through the Trans-Omics for Precision Medicine (TOPMed) program. The genetic ancestry was determined by the CLSA team. Low-quality imputed genetic variants were excluded. Only single nucleotide polymorphisms (SNPs) with a minor allele frequency (MAF) above 0.1%, an imputation quality score greater than 0.3, and a missing rate below 0.1 were retained. This resulted in approximately 15.4 million SNPs available for genome-wide association studies (GWAS). The plasma samples were analysed using the Ultrahigh Performance Liquid Chromatography-Tandem Mass Spectroscopy (UPLC-MS/MS) platform by Metabolon Inc. A total of 1,458 metabolites were quantified. The metabolomics data underwent strict quality control and curation to ensure accurate and consistent identification of true chemical entities. This process eliminated systemic artifacts, misassignments, and background noise. We retained 1,091 metabolites and 309 metabolite ratios that have fewer than 50% missing measurements in the samples. The study was approved by the research ethics boards of the Jewish General Hospital under protocol number 2021-2762.

**The TwinsUK and the Cooperative Health Research in the Region of Augsburg (KORA) study**

The TwinsUK cohort primarily includes adult female twins recruited from the general population in the UK, ranging in age from 17 to 85 years old. Participants in the KORA study come from Augsburg in southern Germany, aged between 32 and 77 years, with an equal number of males and females. Together, these two European population studies encompassed a total of 7,824 adults. In TwinsUK and KORA, participants were genotyped using Illumina HumanHap300/610Q/1M-Duo/1.2MDuo 1M and Affymetrix Genome-Wide Human SNP Array 6.0, respectively. Genetic association analyses were conducted on approximately 2.1 million SNPs estimated from a HapMap2-based genotype dataset. Results from both datasets were combined using an inverse variance meta-analysis adjusted for effect size estimates and genomic control standard errors, and SNPs with low imputation quality (info < 0.4), low minor allele frequency (< 0.01), significant heterogeneity of effects between cohorts (defined as heterogeneity p < 0.001 and p-value≥0.001=0.05/486 in either cohort), or present only in one cohort were removed. The lead SNP for each locus was selected as the SNP with the lowest p-value for any trait at that locus. All associations passing this threshold were assigned to independent loci by iteratively allocating the lead SNP with the lowest p-value and SNPs within 500kb of it to the same locus. A total of 529 metabolites were analyzed using liquid chromatography and gas chromatography separation coupled with tandem mass spectrometry, integrated with gene expression data, heritability estimates, and overlap with known drug targets. After stringent quality controls, a subset of 486 metabolites was available for genetic analysis in both cohorts, including 309 known and 177 unknown metabolites. Participants in the TwinsUK and KORA studies provided written informed consent and were approved by the local ethics committees at Guy’s and St. Thomas’ Hospital and Bayerische Landesärztekammer, respectively.

**The UK Biobank project**

UK Biobank is a population-based health research resource consisting of approximately 500,000 people, aged between 38 years and 73 years, who were recruited between the years 2006 and 2010 from across the UK. Particularly focused on identifying determinants of human diseases in middle-aged and older individuals, participants provided a range of information (such as demographics, health status, lifestyle measures, cognitive testing, personality self-report, and physical and mental health measures) via questionnaires and interviews; anthropometric measures, BP readings and samples of blood, urine and saliva were also taken (data available at www.ukbiobank.ac.uk). UK Biobank received ethical approval from the Research Ethics Committee (REC reference for UK Biobank is 11/NW/0382). This work was carried out using the following diseases record in UK Biobank project: Graves' disease (554 cases and 399,034 controls, phecode-242.1), chronic lymphocytic thyroiditis (249 cases and 399,034 controls, phecode-245.21), and hypothyroidism (18,404 cases and 399,034 controls, phecode-244).

**Prostate cancer studies in the OncoArray project**

The Project of Breast, Ovary, and Endometrial Cancer Association Consortium (http://bcac.ccge.medschl.cam.ac.uk/) is a collaboration involving many groups around the world, focusing on the genetic susceptibility to a specific cancer. The project of cancer association consortium is mainly composed of oncoarray, COGS and other scattered GWAS. BCAC encompasses 78 studies of OncoArray, 52 studies of COGS, and 9 studies of other scattered GWAS. OCAC encompasses 35 studies of OncoArray, 24 studies of COGS, and 4 studies of other scattered GWAS. ECAC encompasses 28 studies of OncoArray, 14 studies of COGS, and 10 studies of other scattered GWAS. Refer to appendix 2~5 for basic information of each series of studies.

**Appendix 2. Prostate cancer studies in the OncoArray project**

| **Study Acronym** | **Study Name** | **Country** | **Controls** | **Cases** |
| --- | --- | --- | --- | --- |
| Aarhus | Australian Breast Cancer Family Study | Australia | 643 | 551 |
| AHS | Amsterdam Breast Cancer Study | Netherlands | 1264 | 1429 |
| BBCC | Bavarian Breast Cancer Cases and Controls | Germany | 564 | 458 |
| BBCS | British Breast Cancer Study | UK | 1507 | 1397 |
| BIGGS | Breast Cancer in Galway Genetic Study | Ireland | 836 | 719 |
| BSUCH | Breast Cancer Study of the University Clinic Heidelberg | Germany | 848 | 954 |
| CECILE | CECILE Breast cancer study | France | 1019 | 999 |
| CGPS | Copenhagen General Population Study | Denmark | 2901 | 4086 |
| CNIO-BCS | Spanish National Cancer Centre Breast Cancer Study | Spain | 902 | 876 |
| CTS | California Teachers Study | USA | 69 | 70 |
| DEMOKRITOS | DEMOKRITOS | Greece | 413 | 95 |
| ESTHER | ESTHER Breast Cancer Study | Germany | 478 | 502 |
| GENICA | Gene Environment Interaction & Breast Cancer in Germany | Germany | 464 | 427 |
| HEBCS | Helsinki Breast Cancer Study | Finland | 1658 | 1233 |
| HMBCS | Hannover-Minsk Breast Cancer Study | Belarus | 690 | 130 |
| KARBAC | Karolinska Breast Cancer Study | Sweden | 722 | 662 |
| KBCP | Kuopio Breast Cancer Project | Finland | 445 | 251 |
| kConFab/ AOCS | Kathleen Cuningham Foundation Consortium for Research into Familial Breast Cancer /Australian Ovarian Cancer Study | Australia | 575 | 897 |
| LMBC | Leuven Multidisciplinary Breast Centre | Belgium | 2671 | 1388 |
| MARIE | Mammary Carcinoma Risk Factor Investigation | Germany | 1796 | 1778 |
| MBCSG | Milan Breast Cancer Study Group | Italy | 488 | 400 |
| MCBCS | Mayo Clinic Breast Cancer Study | USA | 1862 | 1931 |
| MCCS | Melbourne Collaborative Cohort Study | Australia | 614 | 511 |
| MEC | Multiethnic Cohort | USA | 731 | 741 |
| MTLGEBCS | Montreal Gene-Environment Breast Cancer Study | Canada | 489 | 436 |
| NBCS | Norwegian Breast Cancer Study | Norway | 22 | 70 |
| NBHS_TN | Nashville Breast Health Study (Triple Negative) | USA | 125 | 118 |
| OBCS | Oulu Breast Cancer Study | Finland | 507 | 414 |
| OFBCR | Ontario Familial Breast Cancer Registry | Canada | 1175 | 511 |
| ORIGO | Leiden University Medical Centre Breast Cancer Study | Netherlands | 357 | 327 |
| OSU | The Stefanie Spielman Breast Bank and the Columbus Area Control Sample Bank | USA | 207 | 203 |
| PBCS | NCI Polish Breast Cancer Study | Poland | 519 | 424 |
| pKARMA | Karolinska Mammography Project for Risk Prediction of Breast Cancer - prevalent cases | Sweden | 5428 | 5537 |
| RBCS | Rotterdam Breast Cancer Study | Netherlands | 664 | 699 |
| RPCI | Roswell Park Cancer Institute | USA | 136 | 126 |
| SASBAC | Singapore and Sweden Breast Cancer Study | Sweden | 397 | 661 |
| SBCS | Sheffield Breast Cancer Study | UK | 839 | 848 |
| SEARCH | Study of Epidemiology & Risk Factors in Cancer Heredity | UK | 9294 | 8068 |
| SKKDKFZ | Städtisches Klinikum Karlsruhe Deutsches Krebsforschungszentrum Study | Germany | 136 | 168 |
| SZBCS | IHCC-Szczecin Breast Cancer Study | Poland | 365 | 315 |
| UKBGS | Breakthrough Generations Study | UK | 470 | 470 |
| ACP | Asian Cancer Project | Thailand | 423 | 636 |
| HERPACC | Hospital-based Epidemiologic Research Program at Aichi Cancer Center | Japan | 694 | 1376 |
| LAABC | Los Angeles County Asian-American Breast Cancer Case-Control Study | USA | 812 | 990 |
| MYBRCA | Malaysian Breast Cancer Genetic Study | Malaysia | 770 | 610 |
| SBCGS | Shanghai Breast Cancer Genetic Study | China | 848 | 892 |
| SEBCS | Seoul Breast Cancer Study | South Korea | 1162 | 1129 |
| SGBCC | Singapore Breast Cancer Cohort | Singapore | 533 | 502 |
| TBCS | IARC-Thai Breast Cancer | Thailand | 138 | 253 |
| TWBCS | Taiwanese Breast Cancer Study | Taiwan | 889 | 236 |
| SCCS | Southern Community Cohort Study | USA | 679 | 680 |
| NBHS | Nashville Breast Health Study | USA | 437 | 252 |
