# Supplementary material for: Associations between circulating metabolites and pca: a bidirectional two-sample Mendelian randomization study
Source: Discov Oncol. 2025 Jul 18;16:1370. doi: 10.1007/s12672-025-03204-9 (PMC12274190; doi:10.1007/s12672-025-03204-9)
Supplement: Supplementary file 3 — Supplementary Material 3 [file 12672_2025_3204_MOESM3_ESM.docx]

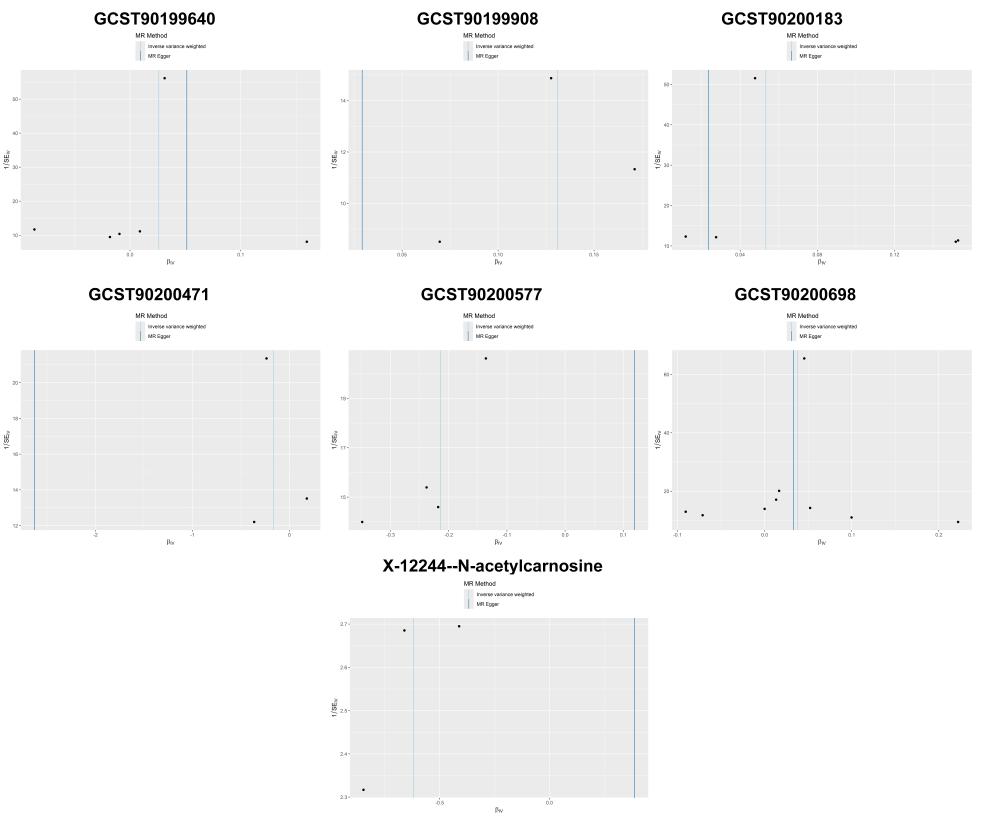


Figure S1 Funnel plot of MR heterogeneity between circulating metabolites and prostate cancer of Oncoarray


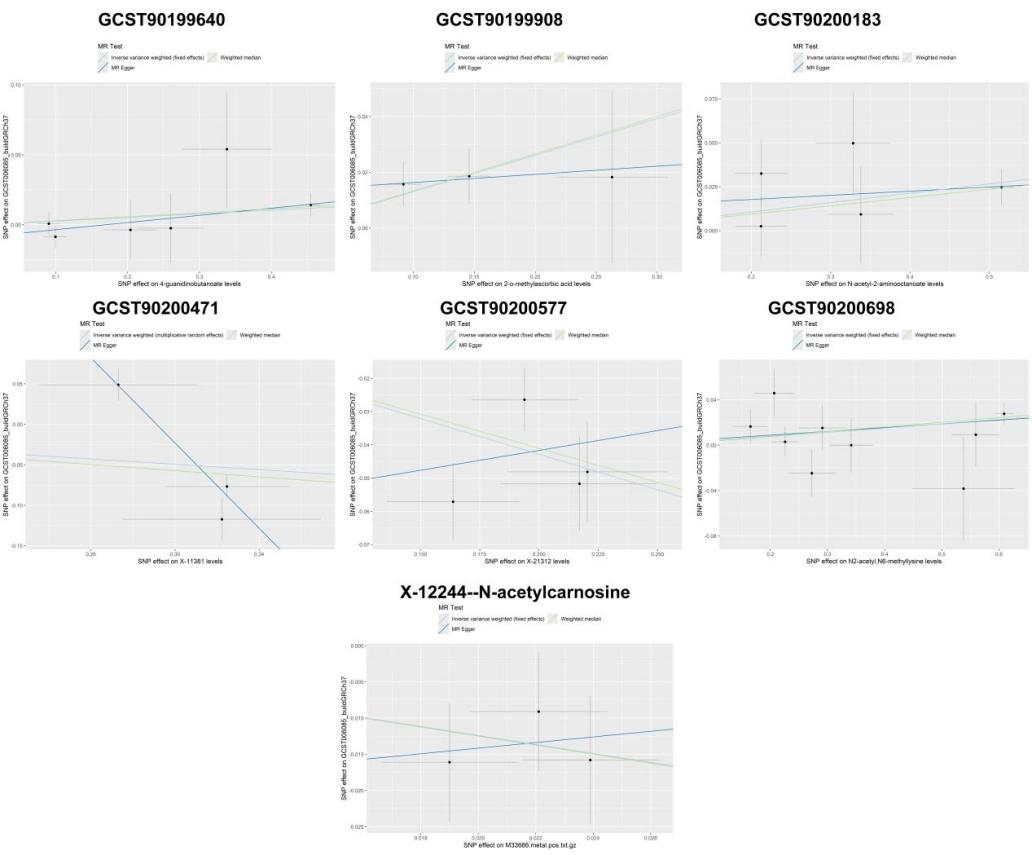


Figure S2 Scatter plot of MR-Egger intercept between circulating metabolites and prostate cancer of Oncoarray


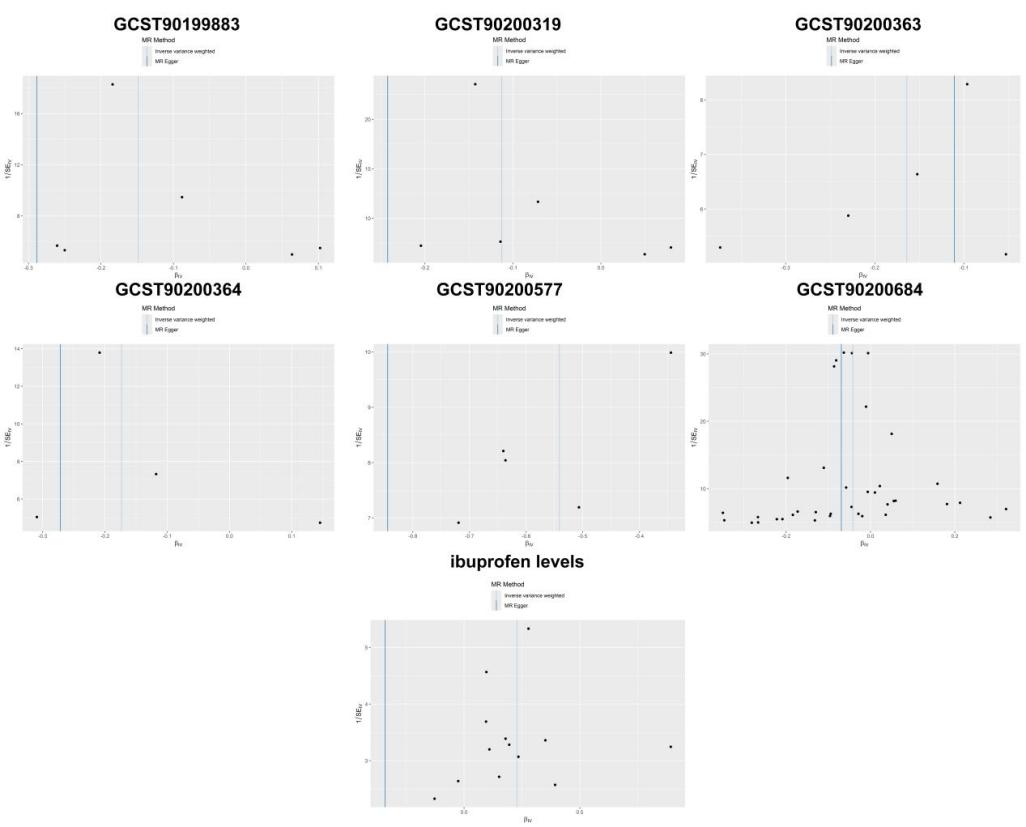


Figure S3 Funnel plot of MR heterogeneity between circulating metabolites and prostate cancer of UK Biobank


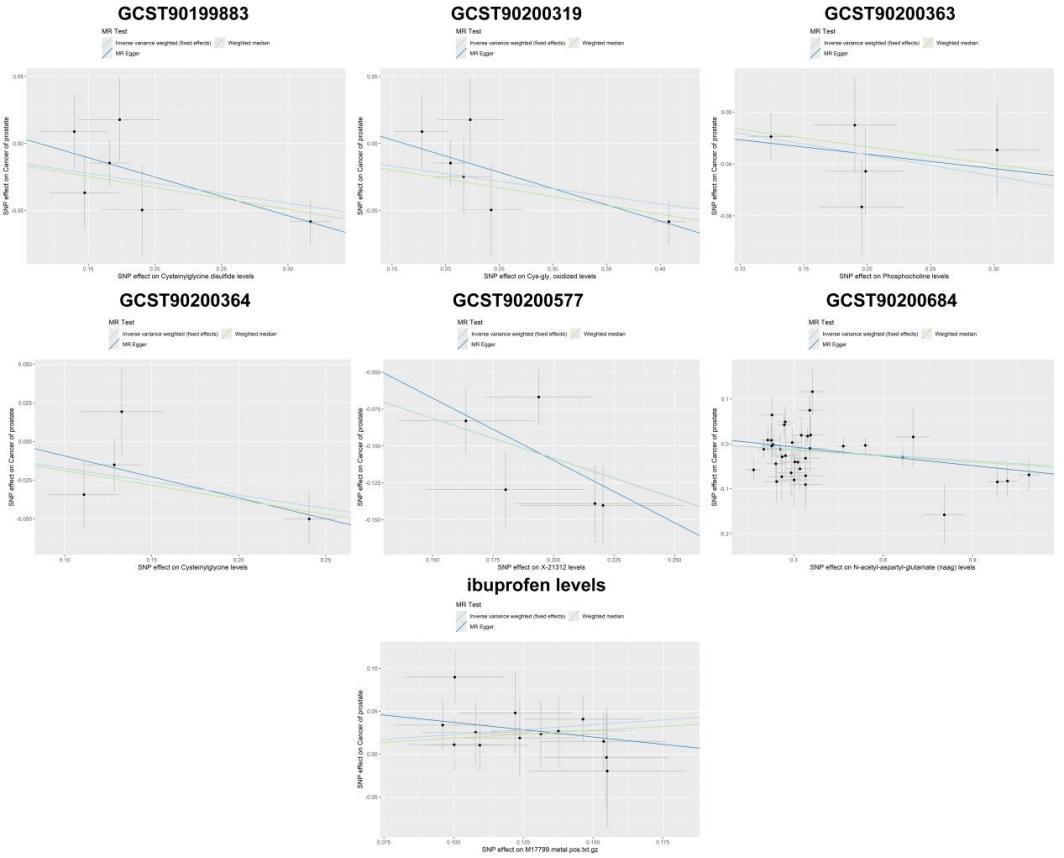


Figure S4 Scatter plot of MR-Egger intercept between circulating metabolites and prostate cancer of UK Biobank


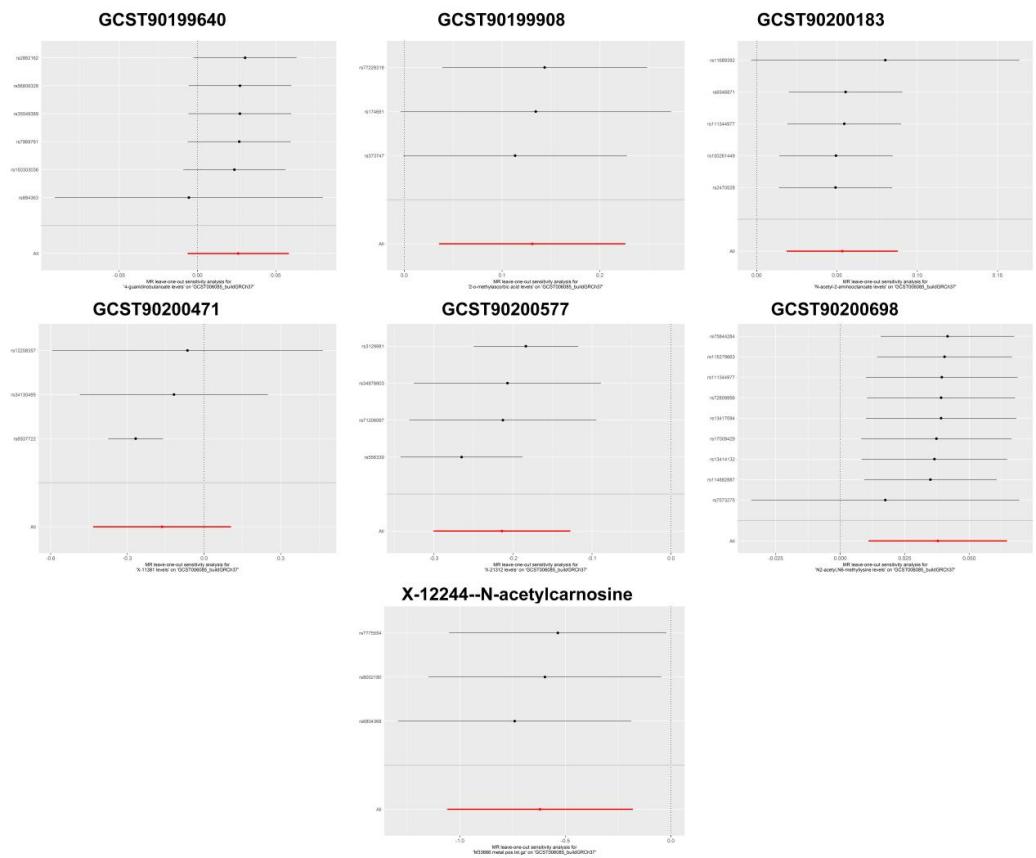


Figure S5 LOO analysis for MR between circulating metabolites and prostate cancer of Oncoarray


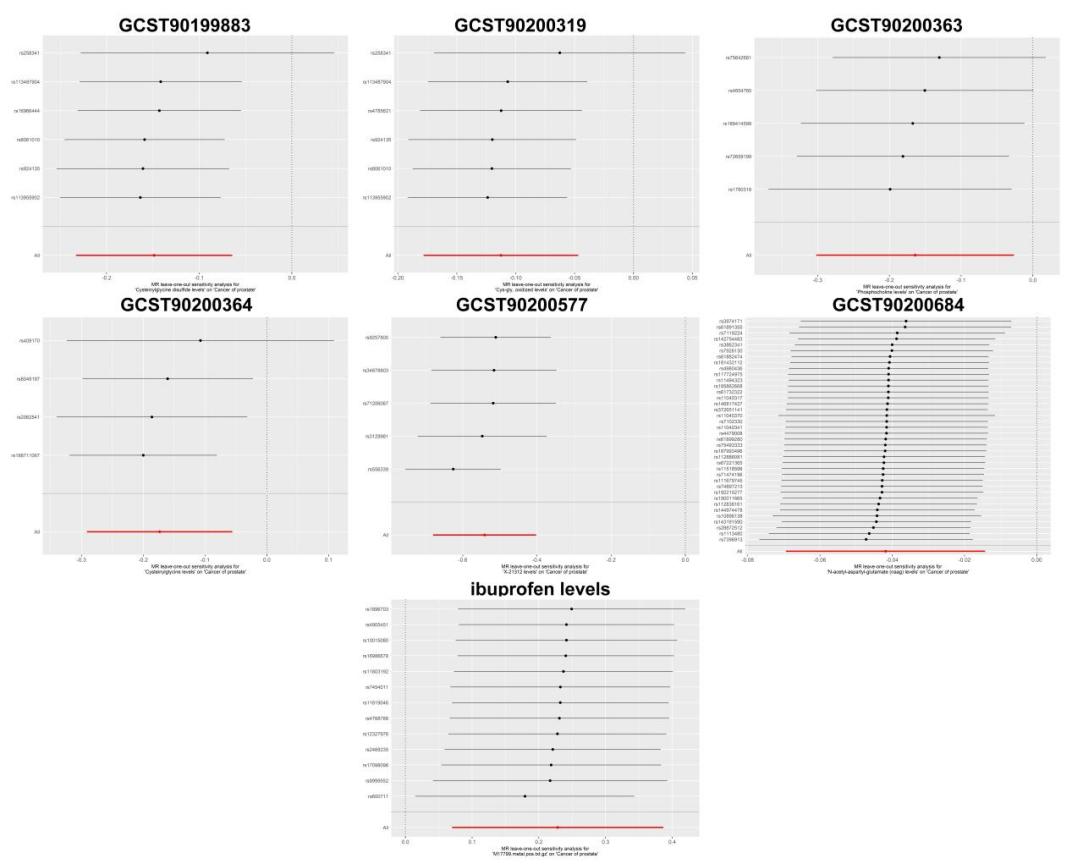


Figure S6 LOO analysis for MR between circulating metabolites and prostate cancer of UK Biobank
